# Optimization of Ultrasonic-Assisted Extraction and Purification of Rhein from *Cassia fistula* Pod Pulp

**DOI:** 10.3390/molecules24102013

**Published:** 2019-05-26

**Authors:** Bancha Yingngam, Haiyu Zhao, Bian Baolin, Nipawan Pongprom, Adelheid Brantner

**Affiliations:** 1 Department of Pharmaceutical Chemistry and Technology, Faculty of Pharmaceutical Sciences, Ubon Ratchathani University, Ubon Ratchathani 34190, Thailand; banchaying@yahoo.com; 2 Institute of Chinese Materia Medica, China Academy of Chinese Medical Sciences, No. 16 Nanxiaojie, Dongzhimen Nei Ave, Beijing 100700, China; hyzhao@icmm.ac.cn (H.Z.); blbian@icmm.ac.cn (B.B.); 3 Department of Chemistry, Faculty of Science, Ubon Ratchathani University, Ubon Ratchathani 34190, Thailand; nipawan.p@ubu.ac.th; 4 Institute of Pharmaceutical Sciences, Department of Pharmacognosy, University of Graz, Universitaetsplatz 4/1, A-8010 Graz, Austria

**Keywords:** *Cassia fistula*, central composite design, extraction, rhein, purification, response surface methodology

## Abstract

Rhein is used as an active ingredient in laxatives in medicinal herbal products and is a chemical marker for quality control purposes. Thus, a simple and effective method for the optimized extraction of a high amount of rhein from the fruit pulp of *Cassia fistula* was investigated using ultrasonic-assisted extraction (UAE). The response surface methodology was applied to find the most suitable parameters for optimizing the extraction process and to study the factors’ relationships with each other. The best conditions for ultrasonic extraction were the application of 1:40 g/mL solid-to-liquid ratio and 10% EtOH–H_2_O as a solvent at 75 °C for 40 min. This method was compared to a conventional decoction in two variations. In these experiments, it was confirmed that the UAE was more favorable than the decoction methods. The resulting crude extract was further purified by liquid–liquid extraction with a basic pH adjustment, followed by recrystallization. High-purity rhein was obtained by using chromatographic techniques and nuclear magnetic resonance spectroscopy. Therefore, this study suggests that UAE is an efficient alternative method for the extraction of rhein from *C. fistula* pod pulp. The resulting optimized conditions can be applied as a useful tool for the large-scale industrial production of a rhein-rich plant extract.

## 1. Introduction

**Figure 1:**
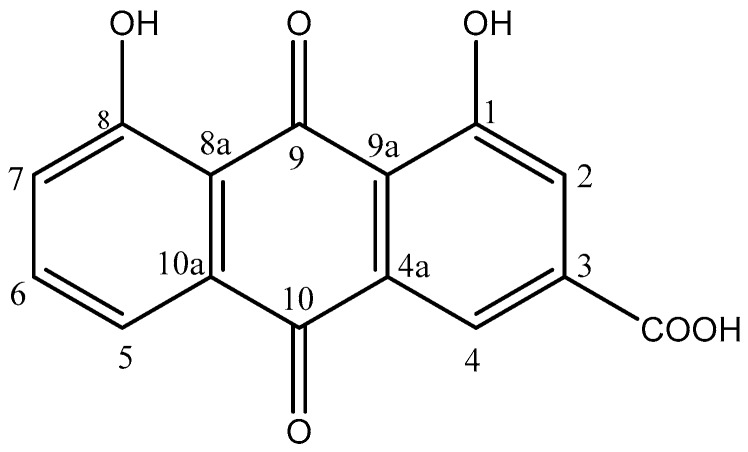
Chemical structure of rhein.

Worldwide, traditional medicine has a long history and contains a vast amount of knowledge regarding the treatment of different diseases. In traditional Thai medicine, as well as Ayurvedic medicine and traditional Chinese medicine, plants are an important part of therapy [1,2,3,4]. *Cassia fistula* L. (Caesalpiniaceae) is a traditional medicinal plant used in Ayurvedic, Thai, and Chinese primary health care as a laxative and to protect against skin mycoses [5,6,7,8]. One of the major ingredients of *C. fistula* is anthraquinone rhein (Figure 1), and its extraction, separation, structural derivation, and total synthesis has been well studied. In 1997, Vittori et al. (United States Patent: 5652265) [9] reported that aloe-emodin derivatives can be used as raw materials to semisynthesize high-purity rhein with good efficiency. Rhein is a traditional Chinese herbal active ingredient that is effective against many diseases [10]. Moreover, it could also be valuable in the treatment of diabetes [11] and inflammation [12]. Rhein is the major ingredient of the fruit pulp of *C. fistula* and is recommended as a chemical marker for the quality control of medicinal products of *C. fistula*. Apart from total synthesis, an alternative way to obtain this pure compound is isolation from natural sources. The fruits of *C. fistula* contain a high amount of rhein as glycosides [7] and can therefore be considered as an essential source for the isolation of rhein. A disadvantage is that the fruits are available only once a year, but they are easily storable. The extract of the pod pulp shows good chemical stability and has a shelf life of 24 months [7]. Consequently, the preparation of rhein-rich plant extracts for the production of herbal medicines or quality control purposes is an important issue.

The traditional method to prepare the extract is to boil the fresh pod pulp of *C. fistula* with water (plant-material-to-solvent ratio 1:10) for 1 h at 95–98 °C [7]. This method has already been compared with other conventional extraction methods such as maceration, percolation, or Soxhlet extraction. Studies have confirmed that traditional decoction is the most suitable method [13]. Modern nonconventional extraction methods, such as ultrasonic-assisted extraction (UAE), have shown beneficial properties in the extraction of various compounds from different plant materials [14,15,16]. One paper described the extraction of five anthraquinones from *Rheum palmatum* L. with UAE [17]. So, this extraction method might also be appropriate for obtaining a high amount of rhein from *C. fistula*.

Besides the extraction process, the separation of rhein from the other ingredients is also of interest. Rhein was isolated from an ethyl acetate extract of the *C. fistula* flowers with column chromatography over silica gel [8]. Presently, there are only a few studies about *C. fistula* in comparison with other plants which contain anthraquinones. Species of *Rheum* have been well examined and different methods have been utilized to extract and isolate the contained anthraquinones [17,18,19]. Liquid–liquid extraction was applied to separate the different anthraquinones from each other from an extract of *Rheum emodi* [20].

The aim of the present work was to find the optimal conditions for the extraction of rhein-rich *C. fistula* extract with UAE. The relevant parameters and final conditions were examined with a central composite design (CCD)-based response surface methodology (RSM). The developed UAE method was compared with the decoction, which is the recommended traditional extraction method. Additionally, rhein was isolated from the extract with liquid–liquid extraction. The carboxylic functional group of rhein was utilized to separate it from the other compounds. Rhein dissociates in the aqueous phase, while the other anthraquinones pass to the organic phase. Characterization was performed by chromatographic techniques, and the chemical structure of rhein was confirmed with ^1^H- and ^13^C-nuclear magnetic resonance (NMR) spectroscopy.

## 2. Results and Discussion

### 2.1. Selection of the Relevant Ranges of the Independent Variables

#### 2.1.1. Plant-Material-to-Solvent Ratio

**Figure 2:**
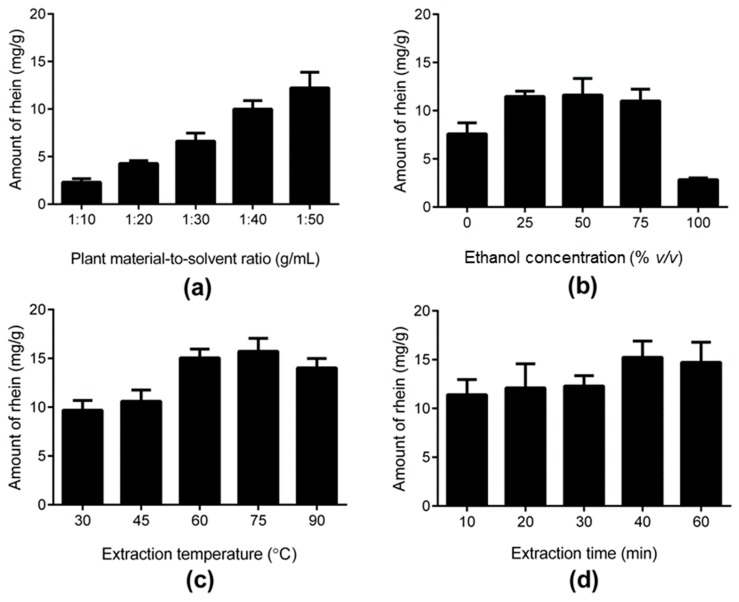
Effects of the independent variables on amount of rhein: (**a**) plant-material-to-solvent ratio, (**b**) ethanol concentration, (**c**) extraction temperature, and (**d**) extraction time. Data are expressed as the means ± SD of three independent experiments.

To determine the effect of the plant-material-to-solvent ratio, 1 g of plant material was mixed with distilled water at ratios of 1:10, 1:20, 1:30, 1:40, and 1:50 g/mL. The samples were extracted for 20 min with UAE. The temperature of the water bath was set at 60 °C. The amount of rhein significantly increased from 2.31 ± 0.36 to 12.22 ± 1.66 mg/g with an altered plant-material-to-solvent ratio from 10 to 50 mL/g (*p* < 0.001) (Figure 2a). This showed that more solvent was necessary to elute rhein from the pod pulp. The tubes had a volume limit of 50 mL. For better handling, the plant-material-to-solvent ratio was set to 1:40 g/mL.

#### 2.1.2. Ethanol Concentration

Ethanol was chosen as the solvent additive for distilled water because of its low toxicity in comparison with other nonpolar solvents [21]. Various ethanol concentrations (0%, 25%, 50%, 75%, and 100% (*v*/*v*) (absolute ethanol)) were chosen for extracting the rhein from *C. fistula*. Forty milliliters of the different ethanol concentrations were added to 1 g of plant material. The samples were placed in an ultrasonic bath for 20 min at 60 °C. The highest amount of rhein (11.48 ± 0.55 mg/g) was detected at a concentration of 25% ethanol, while an increase of the ethanol concentration from 25% to 75% did not lead to a significant increase of the rhein content (Figure 2b). In *C. fistula*, rhein is easy to dissolve in polar solvents such as water. The change in polarity of the solvent generally leads to another composition of the extract [15]. This could be the reason why the amount of rhein dropped to 2.84 ± 0.18 mg/g with an increase of the ethanol concentration from 75% to 100%.

#### 2.1.3. Extraction Temperature

While all other parameters were kept constant, the temperature was varied. The selected values were 30, 45, 60, 75, and 90 °C. The samples were mixed with 40 mL of 25% ethanol in distilled water. The time of UAE application was kept constant at 20 min. The temperature of the water bath varied. The amount of rhein increased as the temperature rose. A soaring rise of 15.05 ± 0.91 mg/g of extracted rhein was detected at 60 °C (Figure 2c). The temperature influences the movement of the molecules. The higher the temperature, the higher the movement speed, which causes, on the one hand, a lower viscosity of the solvent, and on the other hand, a larger diffusion process with better extraction [15]. A temperature change from 60 to 75 °C did not lead to a significantly better result (*p* > 0.05). On the contrary, at 90 °C, a decline of the rhein was detected (*p* < 0.05). The reason for this could be the decomposition of rhein.

#### 2.1.4. Extraction Time

Extraction times of 10, 20, 30, 40, and 60 min were chosen to find the optimal extraction time. The samples were mixed with 40 mL of 25% ethanol in distilled water. The temperature of the water bath was held stable at 60 °C, while the UAE time was modified. In general, a longer extraction duration leads to a higher extract yield because the solvent can penetrate better into the plant material and extract the desired ingredient [14,15]. This experiment revealed a slight rise of extracted rhein within the extraction duration (Figure 2d). The maximum yield of rhein with 15.24 ± 1.67 mg/g was reached after 40 min.

Information from the preliminary investigation was used to specify the conditions of the variables to determine the intermediate, minimum, and maximum values. This was necessary for creating an experimental field with CCD. Therefore, the selected variables for further investigations with CCD were the ethanol concentration, the extraction temperature, and the extraction time. The plant-material-to-solvent ratio was fixed at 1:40 (*w*/*v*) for all further experiments. According to this preliminary exploration, the center points for the different variables were 25% (*v*/*v*), 60 °C, and 25 min. The minimum and maximum values of the ethanol concentrations were set at 0% and 50% (*v*/*v*), the temperature at 35 and 85 °C, and the extraction time at 0 and 50 min.

### 2.2. Fitting the Model

The dependent factor of this research was the amount of rhein (*Y*). The experimental responses were calculated as the amount of rhein in milligrams per gram of plant material. Each experimental run was carried out in quadruplicate and the mean values with standard deviation of each experimental run can be seen in Table 1. The highest amount of rhein was achieved with 15.69 ± 2.24 mg/g of plant material in comparison with the lowest yield of 5.88 ± 1.00 mg/g of plant material. A quadratic regression model was created with the multiple linear regression (MLR) method to use the obtained data from the experiments for prediction purposes. The quadratic regression model obtained from MLR was the following quadratic polynomial equation:

*Y* = 10.36 + 0.43*X*_1_+ 2.44*X*_2_ + 1.44*X*_3_ – 0.47*X*_1_*X*_2_ – 0.68*X*_1_*X*_3_ + 0.65*X*_2_*X*_3_ – 0.08*X*_1_^2^ +0.07*X*_2_^2^ – 0.25*X*_3_^2^. (1)

**Figure 3:**
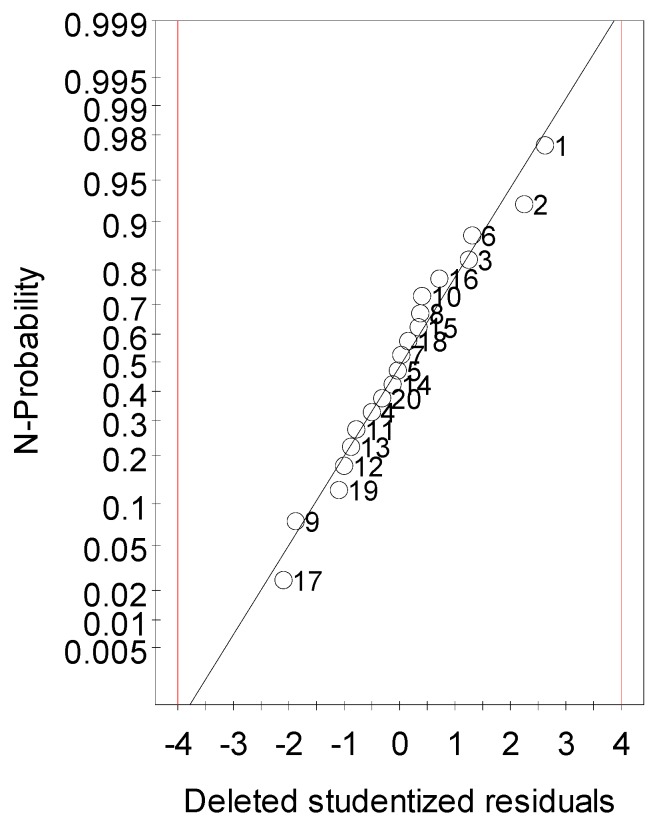
Normal probability plot of the studentized deleted residuals.

The analysis of the adequacy of the fitted model was performed. At first, the residuals of the experimental values were presented graphically in a normal probability plot (N-plot) of studentized deleted residuals to verify the normal distribution (Figure 3). The studentized deleted residuals are the corrected residuals of the estimated standard deviation. The lower the residual value, the higher the precision of the model. The N-plot revealed a normal distribution with a small scatter of some points.

**Figure 4:**
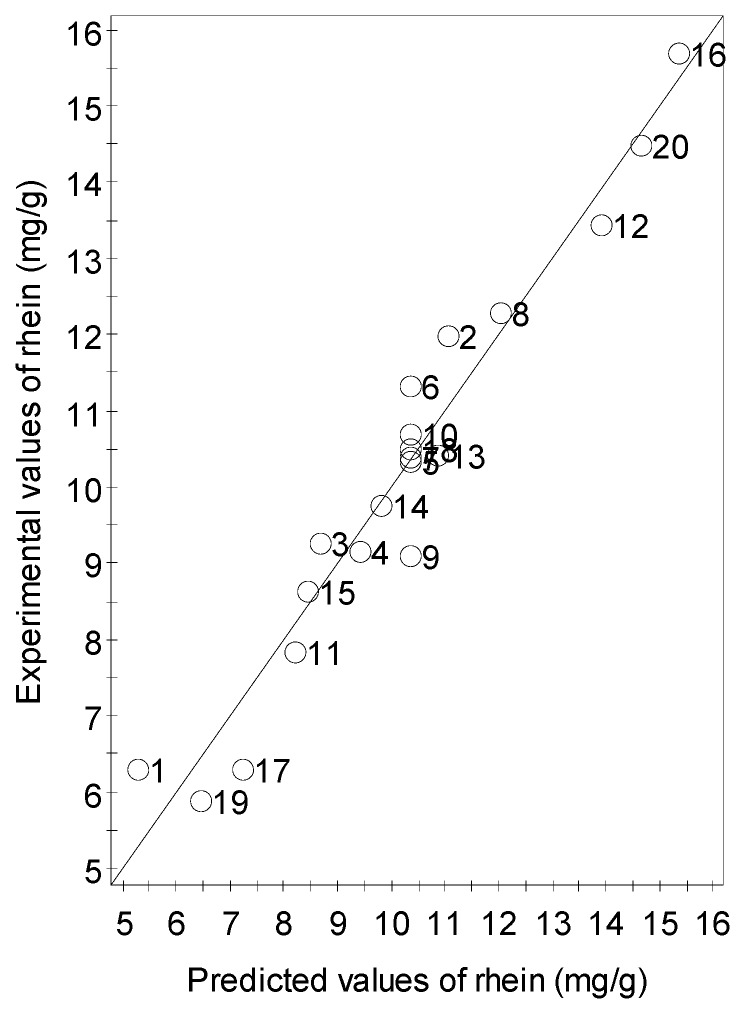
Experimental versus predicted values of rhein amount.

A scatter plot of the relationship between the predicted and experimental values was prepared to evaluate the efficiency of the regression model. The linear correlation of the experimental and predicted values is shown in Figure 4. The experimental values were close to a straight line of the predicted values. An *R*^2^ of 0.9463 indicated a good fit of the quadratic regression model. The more realistic value of 0.8979 of the *R*^2^_adj_ was closely related to *R*^2^. This demonstrated a high correlation between the predicted and observed values. An *R*^2^_pred_ of 0.700 was the lowest acceptable boundary value to produce good predictions with low error value [22]. The difference between *R*^2^_adj_ and *R*^2^_pred_ was less than 0.200, indicating a suitable model for predictions. The developed model was expected to produce 70% of the variability in new experiments. Both graphical figures demonstrated adequate reasoning for the adequacy of the developed regression model.

ANOVA revealed further evidence for the high significance of the regression model with the low *p*-value of <0.0001. This screening was fulfilled because the value was *p* ≤ 0.05. The lack-of-fit compared to the pure error showed no significance (*p* = 0.3102) because the *p*-value was higher than 0.05 (Table 2). Finally, the regression model showed no lack-of-fit and passed all verification. It could be assumed that the polynomial equation of the regression model was appropriate for the purpose of prediction with low predictive error.

The next step was to define the significant factors, which were important for the extraction of rhein from *C. fistula*. The sum of squares of the model (SS_residua_l = 122.09) could be split into the sum of squares of each term to evaluate their impact on the extraction process (Table 2). The extraction temperature (*X*_2_) and time (*X*_3_) showed a highly significant impact on the extracted amount of rhein because of their *p*-values of <0.0001. The *p*-value of the interaction between ethanol concentration and time (*X*_1_*X*_3_) with 0.0442 as well as the interaction between temperature and time (X_2_X_3_) with 0.0525 were near the significance threshold of *p* = 0.050. The ethanol concentration (*X*_1_) of 0.0853 was in the range of *p* = 0.050–0.100, which indicated low significance. The other terms had a *p*-value over 0.100, which indicated that they were insignificant [14,15,22].

### 2.3. Effect of Independent Factors on Rhein Yield

**Figure 5:**
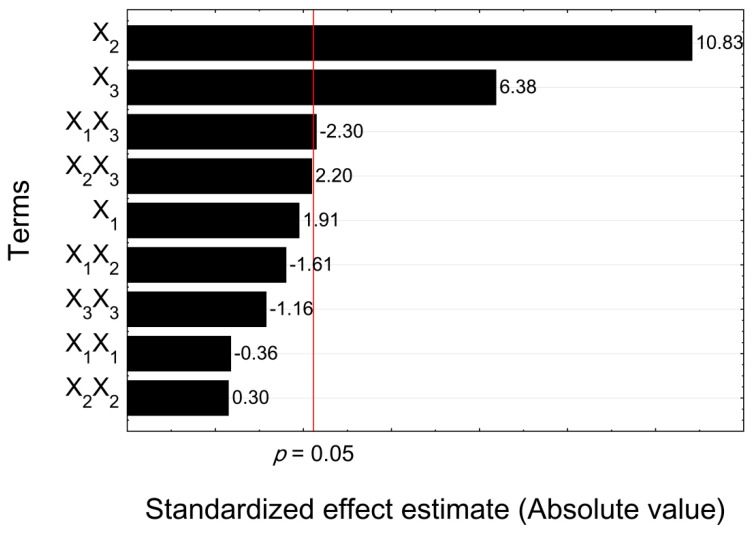
Pareto chart of standardized effects (*X*_1_, ethanol concentration; *X*_2_, extraction temperature; *X*_3_, extraction time).

A way to present the impact or effect of the terms on the extraction process is the Pareto chart (Figure 5). The Pareto chart shows the standardized effects which were calculated from the coded units and their error terms. The coded units referred to dimensionless values which enabled us to compare the different terms. The positive or negative values revealed more information about the impact on the extraction than the *p*-values of Table 2. The terms were sorted by their effect size on rhein extraction. The red line highlights the statistical significance limit (*p* = 0.05). The effects of the temperature (*X*_2_) with +10.83 and time (*X*_3_) with +6.38 were situated far on the right side of this limit, which points out their dominance on the extraction of rhein in comparison with the other terms. The effects of the other terms were negligible and cancelled each other out. This was very interesting in the case of the interaction between ethanol concentration and time (*X*_1_*X*_3_) and between temperature and time (*X*_2_*X*_3_) because the *p*-value of both was close to the limit of *p* = 0.05, which marks the significance threshold. The relationship between the result and the effort was described by Vilfredo Pareto, who established the Pareto principle or 80/20 rule. This principle says that 80% of the results are caused by 20% of the total effort. The results in Figure 5 scientifically support this statement because insignificant differences were observed for other variables and quadratic terms (*p* > 0.05).

### 2.4. Visualization and Interpretation of the Result

**Figure 6:**
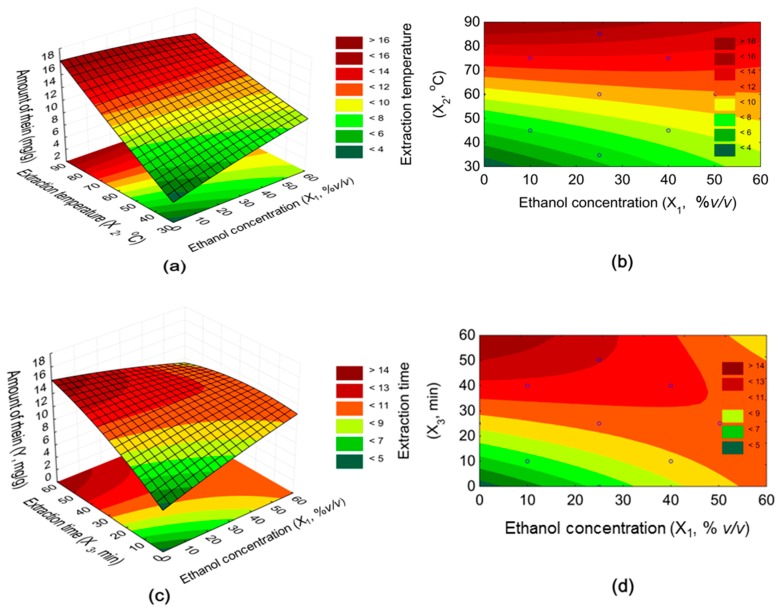

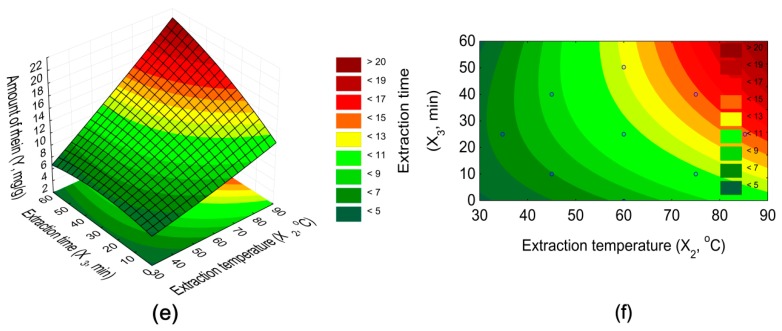
3D response surface and contour plots: (**a,b**) temperatures and ethanol concentrations, (**c,d**) ethanol concentrations and extraction times, and (**e,f**) temperatures and extraction times on amount of rhein.

3D response surface plots and contour plots were created with the developed quadratic polynomial equation in order to visualize the interaction between two independent variables and their effects on the yield of rhein. The quadratic regression model is widely used because of its universal usability in presenting different types of response surfaces [23]. From green to red, the different colors represent the increasing amount of rhein. Figure 6a,b show the effect of ethanol concentration and temperature on rhein extraction when the time is constant. The highest amount of rhein was achieved when the ethanol concentration was low and the temperature was increased to the maximum. While high ethanol concentrations at a lower temperature are an advantage, it turns when the temperature is increased. The reason for this could be the higher solubility of rhein in lower ethanol concentrations at a higher temperature. The conclusion for the interaction between temperature and ethanol concentration is to hold the ethanol concentration low and increase the temperature.

The effect on the yield of the interaction between ethanol concentration and extraction time is shown in Figure 6c,d. Similar to the interaction between ethanol concentration and temperature, the greatest possible amount of rhein was obtained when the extraction time was set high and the ethanol concentration of the solvent was low. When the duration of the extraction was long and the ethanol concentration was over 25% *v*/*v*, the yield gradually decreased. This decrease was stronger than in the interaction between temperature and ethanol concentration.

The 3D response surface of the interaction between temperature and time showed a typical rising ridge (Figure 6e,f). The yield of rhein was positively affected with the rise of both factors, while low values of time and temperature led to a small amount of rhein. These two factors significantly influenced the extraction of rhein from the plant material.

### 2.5. Optimization of UAE for Rhein Isolation and Model Validation

CCD has an integrated optimization tool which facilitates the optimization and proposes different solutions. It must be noted that the optimization of rhein extraction can only be made within the range of the experimental field. The independent factors were held in range, while the aim of the optimization was to maximize the yield of rhein. The recommended solution was a ratio of plant-material-to-solvent of 1:40 g/mL, an ethanol concentration of 10% *v*/*v*, an extraction temperature of 75 °C, and an extraction time of 40 min. The predicted value of rhein (15.34 ± 0.83 mg/g of plant material) was calculated with the developed polynomial equation. Three additional sample batches with 1 g of plant material and a plant-material-to-solvent ration of 1:40 g/mL were extracted by UAE with the recommended solution to verify this prediction. The average of the experimental value amounted to 14.98 ± 0.93 mg/g, which was within the confidence interval of 13.83–16.86 mg/g (*p* > 0.05). The result indicated that the prediction was in accordance with the experimental values and the extraction conditions were optimal to extract a high amount of rhein from *C. fistula*.

As mentioned above, the aim of our study was to optimize the UAE conditions to obtain a rhein-rich plant extract. The independent factors consisted of ethanol concentration, extraction temperature, and time. The solid-to-liquid ratio was fixed at 1:40 g/mL because it gave the highest yield of rhein, and after this level, the rhein yield did not improve. We think that these factors are enough because the ultrasonic frequency used was in the range of a laboratory and pilot-scale ultrasonic bath (working frequency of 45 kHz). The combination of water and ethanol was used as a solvent because of its safety profile for pharmaceutical usage. Thus, the resulting optimized conditions should be enough to demonstrate the optimal extraction mode for rhein.

### 2.6. UAE Compared with the Decoction Method

The next step was to compare the optimized UAE (A) with the classic decoction method (B) and the traditional method (C). The extraction conditions of the different variations are shown in Table 3. The variations A and B were performed under the same extraction conditions. In Thailand, the traditional and recommended method (C) to prepare *C. fistula* pulp is decoction [13]. A study on the extraction of anthraquinones from *C. fistula* pulp with four different conventional extraction procedures concluded that decoction was the most convenient method. The solvent was distilled water. The recommended plant-material-to-solvent ratio was 1:10 *w*/*v* (10 g for 100 mL), with an extraction duration of 60 min and an extraction temperature of 95–98 °C. After the extraction process, the supernatant was separated from the pulp. The pulp was extracted repeatedly and each time the presence of the anthraquinones in the decoctions was tested with the Borntraeger reaction. The decoctions showing a positive Borntraeger reaction were combined. Nine extraction cycles were performed, which lasted 9 h, and the volume of solvent increased to 900 mL. Because of the time factor, variation C was preferred, which included only a single extraction process instead of an exhaustive extraction.

Comparing methods A and B, the obtained amount of rhein from the extraction with UAE (14.98 ± 0.93 mg/g of plant material) was twice as much as the decoction (6.12 ± 0.71 mg/g of plant material) (*p* < 0.001). The increased rhein content is attributed to the different mechanical effects of UAE. The combination of these mechanical effects may enhance the penetration of the solvent into the plant material and cause a higher mass transfer, which leads to an increased solute transfer rate. Also, acoustic streaming and cavitation, which induce high speed jets, positively affect the process of mixing [14,16,17,24].

Most interestingly, the two decoction methods led to different results. The optimized extraction conditions without UAE (variant B) caused an increased rhein yield which was three times higher than the result after applying the traditional decoction method. It is evident that the altered plant-material-to-solvent ratios from 1:10 to 1:50 *w*/*v*, the addition of a small amount ethanol (10% *v*/*v*) to the solvent, and the reduced temperature had a crucial influence on the result, even if the duration of the optimized extraction was shorter. This indicates that the extraction conditions of the traditional method may not be appropriate to extract a high amount of rhein from *C. fistula* pulp and the optimized conditions should be preferred.

In the case of the traditional method (variation C) [13], it is obvious that a single extraction leads to a lower amount of rhein (2.24 ± 0.59 mg/g). However, the traditional extraction method is time consuming, especially when an exhausted extraction is performed. UAE can be an appropriate method to shorten the extraction duration, even if an exhausted extraction is carried out with UAE. This method may also achieve a reduction of solvent consumption [16,24].

### 2.7. Purification and Identification of Rhein

Rhein has already been isolated from the flowers of *C. fistula* by column chromatography. The elution started with the hexane phase and changed into a mixture of hexane and ethyl acetate (95:5 to 0:100) with time. The result was 117 fractions, which were combined into 24 fractions after thin-layer chromatography (TLC) [8]. Another study utilized high-speed countercurrent chromatography with pH-modulated stepwise elution to separate the different anthraquinones from various traditional Chinese plants [25]. The liquid–liquid extraction for separation purposes has already been used to isolate the different anthraquinones from *R. emodi* [26]. This separation method was modified for the isolation of rhein from *C. fistula*.

**Figure 7:**
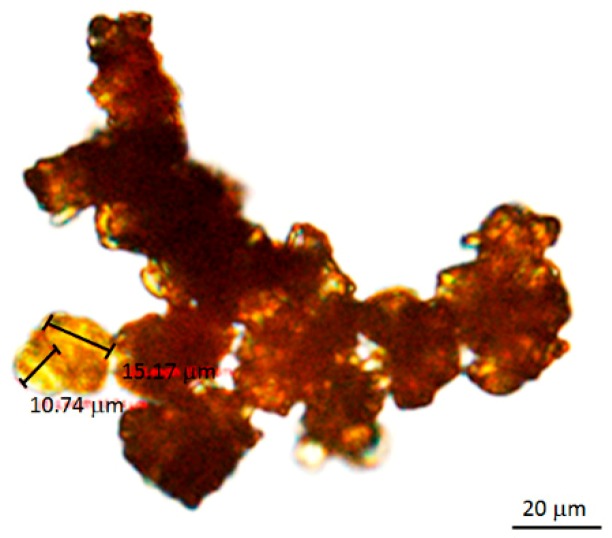
Rhein crystal observed under a light microscope (magnification 20×).

The functional groups in the positions C-3 and C-6 of the 1,8-dihydroxyanthraquinone lead to different chemical characteristics such as acidity or solubility. Rhein has at position C-3 a carboxylic acid group which results in high acidity compared with the other anthraquinones (chrysophanol, aloe-emodin, emodin, and physcion). The carboxylic acid group of rhein exists in an alkaline environment in its ionized form, which is called carboxylate. In this case, the alkaline sodium hydrogen carbonate was added to the aqueous extraction to form the carboxylate. The other anthraquinones were not affected by NaHCO_3_ because they need a stronger alkaline for ionization [25]. The separation was achieved because the ionized rhein dissociated in the aqueous phase, while the other anthraquinones dissolved in the organic phase. At the beginning, the aqueous extract was checked with pH indicator strips and revealed a pH value of 5. The recommended ratio of NaHCO_3_ to aqueous extract was 1:10 (*w*/*w*). An examination with a microscope showed small rhein crystals arranged as agglomerates (Figure 7). The color of the purified rhein was orange-brown. The quantity of the obtained rhein amounted to 325 mg from 30 g of *C. fistula* fruit pulp.

**Figure 8:**
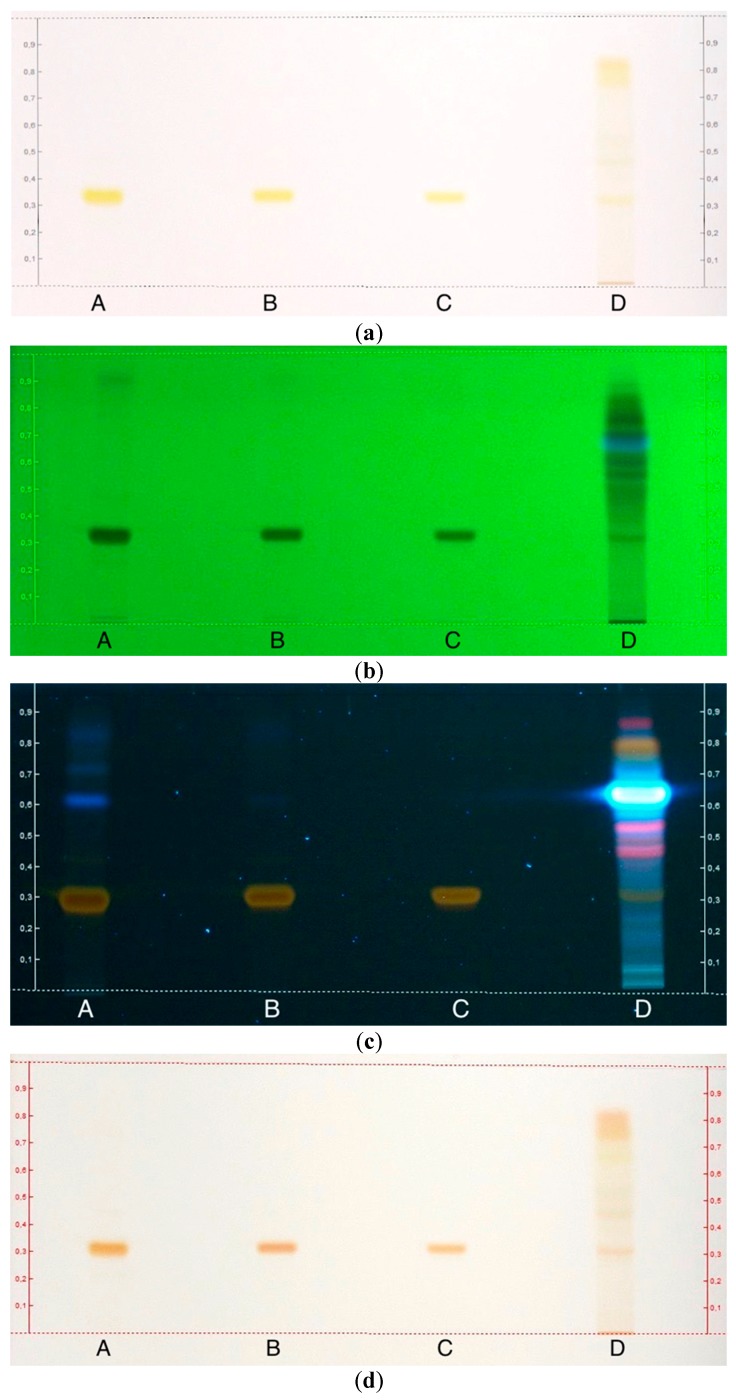
Thin-layer chromatography (TLC) after development at (**a**) visible light, (**b**) 254 nm, (**c**) 366 nm, and (**d**) after spraying with 10% methanolic KOH reagent (A = rhein sample before purification, B = rhein sample after purification, C = rhein standard, and D = residue from the CHCl_3_ layer).

Thin-layer chromatography was used to check the purified rhein. As depicted in Figure 8a, samples A and B showed the same yellow spot as the rhein standard (C) in visible light. All three spots had the same *R_f_* value of 0.32, so the two yellow spots from A and B were identified as rhein. The *R_f_* value of rhein was specified as 0.36 in the literature [27]. The residual from the CHCl_3_ layer (D) revealed various spots from the other anthraquinones and ingredients from the fruit pulp of *C. fistula*. Also, a small spot of rhein was evident. Generally, polar substitutions at a chemical structure lead to a low *R_f_* value, while the *R_f_* value increases with lipophilic substitutions. In the case of anthraquinones, different substitutions at C-3 resulted in the following order on the TLC: COOH < CH_2_OH < CH_3_ < H. Substitutions at position C-6 of the anthraquinone structure affect the order as follows: OH < OCH_3_ < H [28]. This explains why rhein with its polar carboxylic acid showed an *R_f_* value of <0.5, while the other anthraquinones (D) were less polar and moved with the mobile phase.

The same chromatogram was detected under ultraviolet light. At 254 nm (Figure 8b), the rhein spot of samples A–C quenched the fluorescence of the TLC plate, which led to dark spots. Sample D showed other compounds which were separated from rhein. Besides the rhein spot, a light shadow was detected for sample A, which was other compounds and, consequently, impurities. It should be mentioned that no impurities were detected under visible light for sample A. No other compounds were detected after the purification (B) and only a single rhein spot appeared.

The impurities of sample A fluoresced at 366 nm (Figure 8c), while the rhein spots appeared as a beige-brownish color, which was in accordance with the literature [29]. Rhein had an *R_f_* value of 0.32, while the *R_f_* values of the impurities ranged between 0.41 and 0.90. Samples B and C showed a single rhein spot, while D presented all anthraquinones and other substances. Especially, the spot with an *R_f_* value of 0.66 was very dominant, with a bright blue fluorescence.

After the UV–Vis examination, the TLC was sprayed with 10% methanolic KOH reagent to test the Borntraeger reaction. The rhein spots interacted with the spraying reagent and the color changed immediately from yellow to a pinkish color in visible light (Figure 8d).

**Figure 9:**
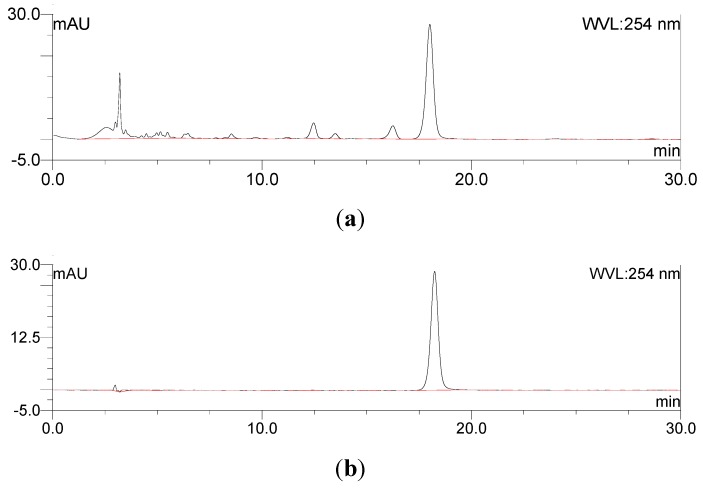
HPLC chromatograms of rhein sample (**a**) before and (**b**) after purification.

**Figure 10:**
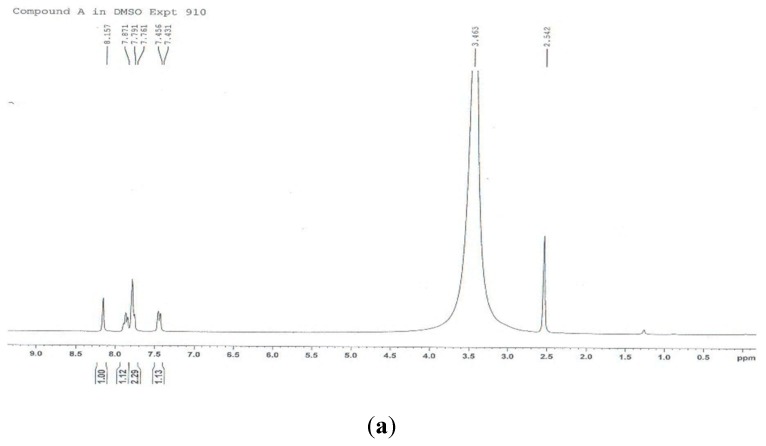

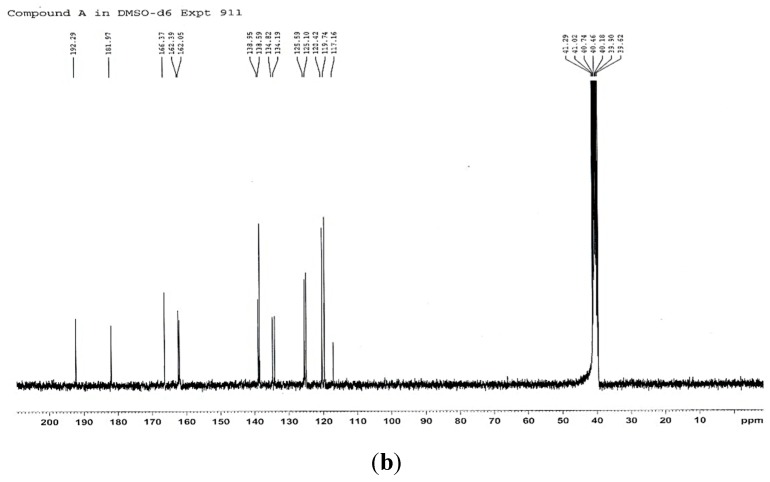
(**a**) ^1^H- and (**b**) ^13^C-NMR spectra of the isolated rhein.

In addition to the TLC, the rhein sample before and after purification was analyzed by HPLC (Figure 9a,b). HPLC is more sensitive than the TLC. The chromatogram was made of the isolated rhein before purification. The signal was detected at 18 min. Besides this large peak, several smaller peaks were detected before. These peaks originated from the impurities. The chromatogram of the rhein after purification showed a large peak at 18 min of high purity (>99%). There was only low noise at the beginning, which probably originated from the injection. The chemical structure was confirmed by ^1^H- and ^13^C-NMR (Figure 10a,b) (m.p.: 321 °C; EI-MS: *m/z* 283 [M − H]^−^; molecular formula: C_15_H_8_O_6_; ^1^H-NMR (300 MHz, DMSO-*d*_6_) δ ppm: 7.44 (1H, d, *J* = 7.5 Hz, H-7), 7.78 (1H, d, *J* = 7.5 Hz, H-5), 7.79 (1H, s, H-2), 7.87 (1H, t, J = 7.5 Hz, H-6), 8.16 (1H, s, H-4); ^13^C-NMR (300 MHz, DMSO-*d*_6_) δ ppm: 117.16 (C_9a_), 119.74 (C_8a_), 119.74 (C_7_), 120.42 (C_2_), 125.10 (C_5_), 125.59 (C_4_), 134.19 (C_4a_), 134.82 (C_10a_), 138.59 (C_6_), 138.95 (C_3_), 162.05 (C_8_), 162.39 (C_1_), 166.37 (3-COOH), 181.97 (C_10_), 192.29 (C_9_)). The result was in agreement with the literature [30]. The results of the TLC and HPLC characterization and the ^1^H- and ^13^C-NMR elucidation led to the conclusion that rhein was successfully isolated with high purity from the fruit pulp of *C. fistula*.

## 3. Materials and Methods

### 3.1. Materials

Dried and mature *C. fistula* fruits were collected in May 2016 in the area of Ubon Ratchathani University, Thailand and were authenticated according to the Thai Herbal Pharmacopoeia [30] by Asst. Prof. Dr. Bancha Yingngam. The voucher specimen was deposited at the Faculty of Pharmaceutical Science, Ubon Ratchathani University (voucher specimen: BCY UBU No. 028). The woody ripe pod was broken with a knife. The septa with the fruit pulp were separated from the seeds and the shell. The collected plant material was stored in plastic bags in a dark place at 25 °C until use. Rhein from Sigma Chemical Co. (St. Louis, MO, USA) was used as reference substance. All applied organic solvents were of HPLC and analytical grade.

### 3.2. UAE

At first, a one-variable-at-a-time methodology was performed. This preliminary exploration was made to select the independent factors with significant impacts on the amount of rhein and to determine the experimental range of each factor. This information was necessary for optimization with RSM [15,31]. For the sample preparation, 1 g of the fruit pulp was weighed in a tube. The established factors of the preliminary study were plant-material-to-solvent ratio (1:10–50 g/mL), ethanol concentration of the eluent (0–100% *v*/*v*), extraction temperature (30–90 °C), and extraction time (10–60 min).

The sample preparation was as follows: UAE was performed with the ULTRAsonik™ 57H model (250 W, C&A Sales Industrial Supplies, USA). The working frequency was set to 45 kHz. The extraction temperature was controlled by an external circulating water bath (TC-500, Brookfield AMETEK, Middleborough, MA, USA). After UAE, the samples were centrifuged with an Eppendorf Centrifuge 5810 R (Hamburg, Germany) for 5 min, 3500 rpm at 30 °C. The supernatants were filtered with a nylon syringe filter (pore size 0.45 µm) and filled into vials. The amount of rhein was determined by HPLC.

### 3.3. Experimental Design

The CCD was used to create the experimental setting and for developing the regression model. The selected variables and ranges were defined by the preliminary exploration. The independent variables were ethanol concentration (*X*_1_), extraction temperature (*X*_2_), and extraction time (*X*_3_). To evaluate and compare the impact or significance of the factor on the result, it was important to convert the real values of the level of each variable into dimensionless values. The coded units -1.68, -1, 0, +1, and +1.68 specify very low, low, intermediate, high, and very high values, respectively (Table 1) [22,23,24,31].

The CCD consisted of eight factorial points, six axial points, and six replicates at the center point. The factorial experiments contained low (-1) and high (+1) values. The very low (-1.68) and very high (+1.68) values were explored by the axial points, while the center points included the intermediate values (0) of the independent variables. The number of center points was fixed at six for good precision for the lack-of-fit test. This resulted in 20 experimental runs. The UAE conditions for each experimental run are listed in Table 1. The dependent factor of this research was the amount of rhein (*Y*). The experimental responses were calculated as the amount of rhein in milligrams per gram of plant material. The optimum condition of UAE should provide the highest yield of rhein. The optimized model was validated at the optimum conditions with three replications. The average experimental response obtained was then compared to the predicted value.

### 3.4. Quantification of Rhein

HPLC analysis was performed on the model Dionex UltiMate™ 3000 UHPLC from Thermo Fisher Scientific Company (Waltham, MA, USA). The software was Chromeleon Version 6.8. The conditions were described in the literature and adopted for this study [32]. The sample (20 μL) was injected into a Hypersil^®^ GOLD C_18_ column (250 × 4.6 mm, 5 μm particle size, Thermo Scientific, Waltham, MA, USA). A mixture of 0.5% acetic acid in water (A) and methanol (B) (40:60, % *v*/*v*) was used as the mobile phase with a flow rate of 1 mL/min. The detection wavelength was set at 254 and 435 nm with a running time of 30 min. The results are presented as the amount of rhein in milligrams per gram of plant material. Values are means ± standard deviation of at least three separate experiments.

### 3.5. Purification and Identification of Rhein

The optimized UAE was utilized to extract rhein from the pod pulp of *C. fistula* (30 g). The mixtures were filtrated with a suction filter (Burke-Waco, San Diego, CA, USA) and a Buechner flask with vacuum (VWR, Darmstadt, Germany). The filtrates showed a dark brown color with a sweetish odor and were combined. The septa with the pod pulp were re-extracted with fresh solvent at the conditions mentioned above. The mixtures were filtrated again, but compared with the first filtrates, the second filtrates showed a very light color and no odor. So, for the isolation of rhein, only the first filtrates were used. The ethanol content of the filtrate was removed with a rotary evaporator (Buechi Rotavapor R-205, Flawil, Switzerland). The temperature of the water bath was 60 °C and the pressure was slowly reduced from 200 to 60 mbar. Finally, 940 mL of the aqueous extract was obtained and used for the liquid–liquid extraction.

At the beginning, the aqueous extract was checked with pH indicator strips from Neutralit^®^ (Merck, Darmstadt, Germany) and revealed a pH value of 5. The recommended ratio of NaHCO_3_ to aqueous extract was 1:10 (*w*/*w*). Solid NaHCO_3_ was added to the aqueous extract and mixed with a magnetic stirrer for 10 min. The aqueous extract was decanted because not all of the NaHCO_3_ was dissolved. The pH was rechecked and showed a pH value of 8.5.

At first, dichloromethane (CH_2_Cl_2_), hexane (C_6_H_14_), and chloroform (CHCl_3_) were tested for their use as the organic phase for the liquid–liquid extraction. The decision was made in favor of CHCl_3_ because the best separation could be achieved. The ratio of the alkaline aqueous extract and the chloroform was 2:1 (*v*/*v*). The extraction procedure was repeated twice (2 × 100 mL). The CHCl_3_ layers were combined (1800 mL) and the solvent was evaporated with a rotary evaporator (475 mbar, 40 °C water bath, Thermo Fisher Scientific, Waltham, MA, USA). The residue was stored for TLC analysis.

The basic aqueous layer was acidified with concentrated hydrochloric acid (HCl) to pH 1. The pH value was checked with a pH meter from Mettler Toledo Five Easy Plus (Greifensee, Switzerland). The acidification led to the hydrolysis of the rhein-8-*O*-glucoside into a rhein and a glucose moiety. Furthermore, the ionized rhein was transferred into un-ionized rhein, which resulted in the precipitation of rhein. The acidified aqueous layer was stored for 48 h in a refrigerator at 4 °C to ensure satisfactory sedimentation of the precipitated rhein. The sedimented rhein was separated from the acidified aqueous layer at first by decanting and then with a suction filter and a Buechner flask with vacuum. The filter paper was washed three times with a small amount of cold water to remove impurities from the obtained rhein. After drying the filter paper, the rhein was extracted three times with 200 mL of ethyl acetate (EtOAc) for 15 min. The EtOAc extracts were combined and evaporated with a rotary evaporator to obtain the purified rhein.

Thin-layer chromatography was performed to identify the rhein. Briefly, 40 mg of each sample was dissolved in 2 mL MeOH. Then, 10 μL of each sample was applied on a silica gel 60F_254_ sheet (10 × 20 cm) with a CAMAG Automatic TLC Sampler (ATS 4, Muttenz, Switzerland). The composition of the mobile phase followed a publication from Sakulpanich and Gritsanapan [27] about the determination of the anthraquinone glycoside content in *C. fistula* leaf extract. The mobile phase was a mixture of EtOAc:MeOH:H_2_O 100:17:13 (*v*/*v*/*v*).

The structure of the isolated rhein was elucidated by ^1^H- and ^13^C-NMR spectroscopy. The instrument was a Bruker Avance 300 spectrometer (Bruker Corporation, Billerica, MA, USA) and was operated at 300 MHz. At first, the isolated rhein was dissolved in dimethyl sulfoxide-*d*_6_ (DMSO-*d*_6_. The spectrum was compared with literature data [8].

### 3.6. Statistical Analysis

The results are expressed as mean ± standard deviation of triplicate measurements (*n* = 3–4). The data obtained from CCD were analyzed by analysis of variance for the polynomial model (linear, quadratic, and interaction terms) and the accuracy of the regression model. A *p*-value < 0.05 was set to be the minimum of statistically significant differences.

## 4. Conclusions

Rhein was successfully extracted and separated from the fruit pulp of *C. fistula* by UAE combined with CCD. The optimum conditions were plant-material-to-solvent ratio 1:40 g/mL with 10% ethanol in water, extraction temperature of 75 °C, and duration of 40 min. With this method, a high amount of rhein could be obtained in the extract in a short time and with less solvent compared with decoction. The results of this study should serve as the basis for further examinations of rhein extraction from the fruits of *C. fistula* with UAE at a larger scale. From the industrial point of view, the method described in this paper is promising for the production of an extract with a high yield of rhein and may lead to economic advantages for the pharmaceutical industry.

## Figures and Tables

**Table 1:** Amount of rhein and the corresponding experimental runs.

Run	Space Type	Ethanol Concentration (*X*_1_, % *v*/*v*)	Extraction Temperature (*X*_2_, °C)	Extraction Time (*X*_3_, min)	Amount of Rhein (*Y*, mg/g pulp) ^1^
1	Factorial	10	45	10	6.28 ± 1.53
2	Factorial	40	75	10	11.99 ± 1.33
3	Factorial	40	45	40	9.26 ± 1.50
4	Axial	0	60	25	9.14 ± 1.57
5	Center	25	60	25	10.33 ± 2.47
6	Center	25	60	25	11.33 ± 2.48
7	Center	25	60	25	10.38 ± 1.07
8	Axial	25	60	50	12.27 ± 0.24
9	Center	25	60	25	9.09 ± 3.55
10	Center	25	60	25	10.68 ± 1.13
11	Factorial	10	45	40	7.83 ± 0.39
12	Factorial	40	75	40	13.42 ± 1.57
13	Axial	50	60	25	10.41 ± 0.59
14	Factorial	10	75	10	9.75 ± 1.07
15	Factorial	40	45	10	8.62 ± 1.32
16	Factorial	10	75	40	15.69 ± 2.24
17	Axial	25	60	0	6.28 ± 0.50
18	Center	25	60	25	10.49 ± 1.79
19	Axial	25	35	25	5.88 ± 1.00
20	Axial	25	85	25	14.49 ± 2.12

^1^ Data are expressed as means ± SD (n = 4).

**Table 2:** ANOVA of the fitted quadratic polynomial model.

Source	Sum of Squares	Degrees of Freedom	Mean Square	*F*-Value	*p*-Value Probability > *F*
Model	122.09	9	13.57	19.56	<0.0001
*X*_1_	2.53	1	2.53	3.65	0.0853
*X*_2_	81.39	1	81.39	117.37	<0.0001
*X*_3_	28.23	1	28.23	40.70	<0.0001
*X*_1_ *X*_2_	1.80	1	1.80	2.60	0.1378
*X_1_X_3_*	3.67	1	3.67	5.30	0.0442
*X_2_X_3_*	3.35	1	3.35	4.84	0.0525
*X*_1_^2^	0.09	1	0.09	0.13	0.7289
*X*_2_^2^	0.06	1	0.06	0.09	0.7671
*X*_3_^2^	0.94	1	0.94	1.35	0.2721
Residual	6.93	10	0.69		
Lack of Fit	4.26	5	0.85	1.60	0.3102
Pure Error	2.67	5	0.53		
Cor Total	129.03	19			
*R* ^2^	0.9463				
*R* ^2^_adjusted_	0.8979				
*R* ^2^_pred_	0.7000				

**Table 3:** The conditions and results of the different extraction modifications.

Variations	Extraction Method	Plant-Material-to-Solvent Ratio (g/mL)	Ethanol (% *v*/*v*)	Extraction Temp. (°C)	Extraction Time (min)	Amount of Rhein (mg/g pulp) ^1^
A	UAE	1:40	10	75	40	14.98 ± 0.93
B	Decoction	1:40	10	75	40	6.12 ± 0.71
C	Decoction	1:10	0	96	60	2.24 ± 0.59

^1^ Data are expressed as means ± SD (n = 3).
